# Digital transcultural psychiatry in times of crisis: the Help for helpers webinar series

**DOI:** 10.1192/j.eurpsy.2023.39

**Published:** 2023-07-19

**Authors:** M. Rojnic Kuzman

**Affiliations:** ^1^ Zagreb University Hospital Centre; ^2^Zagreb School of Medicine, Zagreb, Croatia

## Abstract

**Abstract:**

The COVID-19 pandemics brought numerus changes in the European mental health systems. One of the major, was the widespread introduction of digital psychiatry across the globe, as the only possible option to maintain the psychiatric care. On February 28th 2022, the European Psychiatric Association has started a network of solidarity for Ukraine to respond to the needs of people in Ukraine as verbalized by the Ukrainian mental health professionals, but also to the need of surrounding countries where people from Ukraine fled to. As verbalized by the colleagues from Ukraine and surrounding countries, one of the unmet educational needs was the education for first line helpers and volunteers from Ukraine and countries surrounding Ukraine where displaced persons fled to. This resulted in the series of webinars on the topics detected as unmet needs (what is normal response to trauma, how to triage dispaced persons in need of help, how to provide first psychological help, how to approach to children of different ages, how to take care of one-self and what is the role of supervision). The webinars were delivered by experienced clincians, trauma experts and experts with lived experince in the war zones, incliding the ones from Ukraine. These are available freely at the EPA website https://www.europsy.net/resource-page/, in several languages.

**Disclosure of Interest:**

None Declared

